# Anti-Xa activity and hemorrhagic event: isn’t it time to consider time ?

**DOI:** 10.1186/s13054-021-03612-7

**Published:** 2021-06-14

**Authors:** Sébastien Redant, Xavier Beretta-Piccoli, Patrick M. Honore, David De Bels, Dominique Biarent

**Affiliations:** 1 Intensive Care Medecine, ICU Department, Hopital Universitaire Des Enfants Reine Fabiola HUDERF, Université Libre de Bruxelles, ULB, Av J.J. Crocq 15, 1020 Brussels, Belgium; 2 ICU Department, Centre Hospitalier Universitaire Brugmann – Brugmann University Hospital, Place Van Gehuchtenplein,4, 1020 Brussels, Belgium

We read with great interest the publication by Descamps et al. which showed that in patients treated with extracorporeal membrane oxygenation (ECMO), mean anti-Xa activity was an independent risk factor for bleeding complications [1]. A non-negligible number of bleeding episodes (i.e., 29%) were observed for anti-Xa values at the lower limit of the official recommendations of 0.3 to 0.7 IU/ml [2]. We were surprised to see that the anti-Xa median value at the time the patients bled was equal to anti-Xa median value measured during the entire total treatment period of all patients who did not bleed. When comparing maximum anti-Xa median values in both groups, no statistical significance was observed; hence, another factor may need to be considered.

In the univariate analysis, the duration of treatment with ECMO was significantly longer in patients who bled. This time factor was not included in the multivariate analysis. We wonder whether a longer exposure to an effective anticoagulation treatment does not expose to a higher risk of bleeding (due to, e.g., manipulations, physiotherapy, sudden blood pressure variations, etc.)?

Karkouti et al. in its prospective study studying transfusion risk in cardiac surgery under cardiopulmonary bypass (CPB) compared 476 patients who received a massive transfusion with 6175 control patients who were not transfused. Massive blood transfusion (MBT) was defined by a transfusion greater than or equal to 5 units of concentrated red blood cells in the 24 h after surgery. A significant difference in the duration of cardiopulmonary bypass (CPB) (144 ± 68 min) was observed in MBT patients versus 98 ± 33 min in not transfused patients (*p* < 0.0001). CPB duration was an independent factor of MBT risk (OR 1.02, *p* < 0.0001with a linear correlation between CPB duration and MBT [3].

In addition, a comparison between group values of anti-thrombin III which play a role in the effectiveness of heparin may be useful to understand factors influencing the occurrence of bleeding [4].

## Data Availability

Not applicable.

## Abbreviations

ECMO: Extracorporeal membrane oxygenation
MBT: Massive blood transfusion
CPB: Cardiopulmonary by pass
