# Supplementary material for: Anti-Xa activity and hemorrhagic event: isn’t it time to consider time ?
Source: Crit Care. 2021 Jun 14;25:210. doi: 10.1186/s13054-021-03612-7 (PMC8201838; doi:10.1186/s13054-021-03612-7)
Supplement: Related texts

# Reply: Response to: Anti-Xa activity and hemorrhagic event: isn’t it time to consider time?

**Authors:** Richard Descamps, Marc-Olivier Fischer, Damien Du Cheyron

**Affiliations:** 3 grid.411149.8 0000 0004 0472 0160 Department of Anesthesiology and Critical Care, Caen University Hospital, 14000 Caen, France; 4 grid.411149.8 0000 0004 0472 0160 Department of Medical Intensive Care, Caen University Hospital, 14000 Caen, France

To the Editor,

We greatly thank Redant et al. for their relevant comments on our article, addressing different issues. To explain the absence of difference between the anti-Xa median value of the non-bleeding group and the anti-Xa median value at the time of bleeding, the delay between the onset of the bleeding (including mostly internal hemorrhagic events such as intracerebral hemorrhage, hemothorax, pericardial or gastrointestinal bleeding (63% of bleedings in our cohort)) and its diagnosis must be pointed out, and may induce an underestimated anti-Xa value at the time of diagnosis. Some patients of the bleeding group may have had their heparin stopped at the time of testing. Regarding the maximum value of anti-Xa, we believe there might a misinterpretation of the results since it was higher in the bleeding group in univariate analysis (*p* = 0.05). This variable was not tested in the multivariate model to avoid confusion bias related to its dependence with the mean anti-Xa values. Of note, the absence of association between max anti-Xa value and bleeding was already reported [5].

We agree that the association between the time of exposure to extracorporeal membrane oxygenation (ECMO) and bleeding must be assessed. A Cox proportional hazards regression analysis adjusted for SAPS II, duration of treatment with ECMO before bleeding, associated IABP/Impella was performed for risk of bleeding under ECMO and mean Anti-Xa. Survival time was duration of ECMO treatment, and the dependent variable was the occurrence of bleeding event. In this model, only SAPS II score (HR, 5.7; 95%CI, 1.01–1.05; *p* = 0.02) and mean Anti-Xa (HR, 14.9; 95%CI, 3.9–69.6; *p* = 0.0001) were associated with bleeding. Interestingly, duration of treatment with ECMO was not identified as independent risk factor of bleeding.

We think cardiac surgery patients are different for several reasons: higher doses of heparin, associated hypothermia, air / blood interface, greater degree of hemodilution, absence of pulsatility and reversal of anticoagulation at the end of surgery [6]. Cardiopulmonary bypass and ECMO populations are therefore not comparable.

Unfortunately, antithrombin data was not available due to the retrospective nature of the study. We agree that this variable may be useful. The benefit of antithrombin monitoring and supplementation in ECMO needs further studies since the use of antithrombin may not change the risk of bleeding events whereas its deficiency was recently associated with an increased risk of thrombosis [7].
